# Impacts of pringle maneuver on hepatectomies: analysis of survival and clinical effects

**DOI:** 10.1590/0102-67202025000053e1922

**Published:** 2026-02-23

**Authors:** Allan Rubens Zucolotto CANSI, Jhonatan de Souza VITOR, João Felipe da Silva LOPES, Rogério Dardengo GLÓRIA

**Affiliations:** 1 Hospital Evangélico de Cachoeiro de Itapemirim, Department of Hepatobiliopancreatic Surgery – Cachoeiro de Itapemirim (ES), Brazil.

**Keywords:** Hepatectomy, Liver, Survival Analysis, Blood Loss, Surgical, Hepatectomia, Fígado, Análise de Sobrevida, Perda Sanguínea Cirúrgica

## Abstract

**Background:**

The Pringle maneuver remains a widely used technique in hepatic surgery with varying opinions on its effects on postoperative outcomes and survival, requiring evidence-based evaluation of its impact on liver function and long-term results.

**Aims:**

The aim of this study was to evaluate the impact of the intermittent Pringle maneuver on postoperative liver function and survival in hepatectomy patients, focusing on early dysfunction markers as prognostic factors.

**Results:**

In this retrospective cohort of 198 patients (106 women and 92 men; mean age, 59 years), the Pringle group showed longer surgical times (226.87±82.18 vs. 184.00±80.90 min, p<0.001) and extended intensive care unit stays (4.02±2.1 vs 3.11±1.9 days, p=0.026), but lower bilirubin levels (2.18±0.33 vs. 3.13±0.39 mg/dL, p=0.049). Multivariate analysis revealed that the Pringle maneuver reduced mortality risk (hazard ratio [HR]=0.540, 95% confidence interval [95%CI]: 0.333–0.876, p=0.013). Early liver dysfunction markers strongly predicted worse outcomes: elevated bilirubin nearly doubled mortality risk (HR 1.975, 95%CI 1.100–3.545, p=0.023), and decreased prothrombin activity tripled it (HR 3.055, 95%CI 1.839–5.075, p<0.001).

**Conclusions:**

While the Pringle maneuver extends operative time and intensive care unit stay, it demonstrates a protective effect on survival. Early postoperative liver dysfunction strongly predicts poor outcomes, emphasizing the importance of careful postoperative monitoring regardless of vascular control strategy. These findings suggest that a controlled intermittent Pringle maneuver offers survival benefits when properly timed.

## ARTICLE HIGHLIGHTS

The intermittent Pringle maneuver reduced mortality risk by 46% in hepatectomy patients, despite longer operative times.

Early postoperative liver dysfunction markers (bilirubin >2 mg/dL or prothrombin activity <50%) strongly predicted poor outcomes and increased mortality risk up to threefold.

Optimal Pringle duration of 16–30 min was associated with favorable survival outcomes and shortest operative times.

Careful postoperative monitoring of liver function remains essential regardless of the vascular control strategy used.

## Summary

**CENTRAL MESSAGE:**

Hepatic ischemia-reperfusion resulting from the Pringle maneuver can induce cellular and tissue damage, potentially leading to postoperative hepatic dysfunction. Moreover, portal congestion caused by the maneuver may lead to bacterial translocation and endotoxemia, potentially contributing to systemic complications. The duration and type of clamping (continuous or intermittent) are critical factors influencing the impact of the Pringle maneuver on postoperative outcomes. However, when properly timed with standardized protocols, the intermittent Pringle maneuver may offer protective effects on long-term survival.

**PERSPECTIVES:**

In this retrospective cohort of 198 hepatectomies, intermittent Pringle maneuver using standardized 15-min ischemia/5-min reperfusion cycles reduced mortality risk by 46% (hazard ratio=0.540, p=0.013), despite longer operative times and increased intensive care unit stays. Early postoperative liver dysfunction emerged as a powerful predictor of poor outcomes, with elevated bilirubin nearly doubling mortality risk and decreased prothrombin activity tripling it. These findings underscore the importance of meticulous postoperative monitoring of hepatic function markers to identify high-risk patients who may require intensified interventions. Future prospective multi-institutional trials should validate optimal Pringle protocols tailored to specific pathologies and patient profiles.

## VISUAL ABSTRACT

**Figure:**
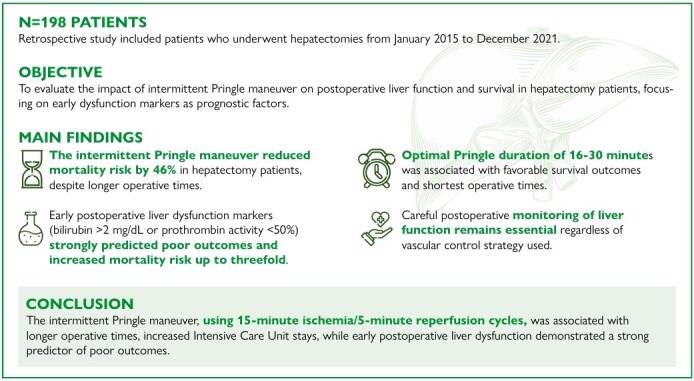

## RESUMO

**Racional:**

A manobra de Pringle é amplamente utilizada em cirurgias hepáticas, mas seus efeitos nos desfechos pós-operatórios e na sobrevida ainda são motivo de debate. Este estudo visa avaliar seu impacto na função hepática e nos resultados em longo prazo.

**Objetivos:**

Avaliar o impacto da manobra de Pringle intermitente na função hepática pós-operatória e na sobrevida em pacientes submetidos à hepatectomia, com enfoque em marcadores precoces de disfunção como fatores prognósticos.

**Resultados:**

Nesta coorte retrospectiva com 198 pacientes (106 mulheres e 92 homens; média de idade: 59 anos), o grupo Pringle apresentou maior tempo cirúrgico (226,87±82,18 vs. 184,00±80,90 minutos, p<0,001) e maior tempo de UTI (4,02±2,1 vs. 3,11±1,9 dias, p=0,026), porém com menores níveis de bilirrubina (2,18±0,33 vs 3,13±0,39 mg/dL, p=0,049, p<0.05). A manobra reduziu o risco de mortalidade (HR 0,540, IC95% 0,333–0,876, p=0,013, p<0.05). Bilirrubina elevada aumentou o risco de morte (HR 1,975, IC 95%: 1,1003,545, p=0,023) e atividade de protrombina <50% o triplicou (HR 3,055, IC95% 1,839–5,075, p<0,001).

**Conclusões:**

Apesar de aumentar o tempo operatório e a permanência em UTI, a manobra de Pringle demonstrou efeito protetor na sobrevida. Disfunção hepática precoce é fortemente associada a piores desfechos, reforçando a necessidade de monitoramento rigoroso no pós-operatório.

## INTRODUCTION

 The Pringle maneuver, pioneered by J. Hogarth Pringle in 1908, has become a cornerstone in hepatic surgery for controlling hemorrhage during resections. This technique involves temporary occlusion of hepatic blood flow by clamping the hepatoduodenal ligament, which houses the hepatic artery and portal vein. While efficacious in reducing intraoperative blood loss, the Pringle maneuver is not without risks and may lead to complications^11 ,17^. 

 Hepatic ischemia-reperfusion resulting from the Pringle maneuver can induce cellular and tissue damage, potentially leading to postoperative hepatic dysfunction. Animal studies have demonstrated that prolonged occlusion of hepatic blood flow may result in oxidative stress, release of inflammatory cytokines, and increased cellular apoptosis^1, 2^. Moreover, portal congestion caused by the maneuver may lead to bacterial translocation and endotoxemia, potentially contributing to systemic complications^6,13^. 

 The duration and type of clamping (continuous or intermittent) are critical factors influencing the impact of the Pringle maneuver on postoperative outcomes. Randomized clinical trials comparing different clamping approaches have shown variable results^3,5^. While some studies suggest that intermittent clamping may be better tolerated than continuous clamping, others have found no significant differences in terms of postoperative complications^4,5,12,14^. 

 Recent meta-analyses have demonstrated significant findings regarding the impact of Pringle maneuver on postoperative outcomes. A comprehensive analysis of 7,480 patients showed that intermittent clamping offers advantages over continuous occlusion, particularly in reducing blood loss without compromising long-term oncological outcomes^9^ . A 2023 meta-analysis of 3,268 patients with hepatocellular carcinoma confirmed that the intermittent Pringle maneuver effectively controls blood loss without affecting long-term survival^8^ . 

 The correlation between early postoperative liver dysfunction and survival has been increasingly recognized in high-impact studies, with evidence showing that patients developing postoperative liver dysfunction exhibit significantly poorer diseasefree survival compared to those without hepatic complications^15^ . Multicenter analyses have identified critical biochemical markers for early detection, demonstrating that prothrombin time (PT) and serum total bilirubin levels on postoperative day 5 are strong predictors of mortality, with PT >1.68 INR (International Normalized Ratio) and bilirubin >4.0 mg/dL indicating high-risk thresholds^18^ . These findings align with studies showing that early-phase PT-INR=1.3 predicts poor prognosis in acute liver injury, while recovery to <1.3 by day 8 correlates with improved survival^10^ . Additionally, the combination of albumin-bilirubin (ALBI) scores and PT enhances predictive accuracy for postprocedural hepatic decompensation^16^ . 

 While modern surgical advancements have enhanced perioperative care, the clinical implications of vascular occlusion techniques on recovery metrics and survival following hepatic resections continue to be debated. In this context, the present study aims to evaluate the impact of the intermittent Pringle maneuver on postoperative outcomes and long-term survival in patients undergoing hepatectomies, with a special focus on early markers of liver dysfunction as prognostic factors. 

## METHODS

### Study design and population

 This retrospective study included patients who underwent hepatectomies from January 2015 to December 2021. All operations were executed by a single experienced surgeon following standardized protocols. A total of 201 consecutive patients underwent elective hepatic resections during the study period. Three patients were excluded due to missing data regarding Pringle maneuver application. The final analysis included 198 consecutive patients who underwent hepatic resections. Sample sizes varied across specific analyses due to missing data: 169 patients were included in the overall survival analysis (29 excluded due to incomplete follow-up), 156 patients in the bilirubin-related analysis (42 excluded due to missing postoperative bilirubin measurements within the first 7 days), and 164 patients in the prothrombin activity-related analysis (34 excluded due to missing postoperative prothrombin measurements within the first 7 days). 

 Missing data were confirmed to be missing at random through sensitivity analysis. The complete-case analysis approach was used for each specific outcome to maintain data integrity while maximizing the use of available information for each specific analysis, rather than employing imputation methods. The choice to use the maneuver was based on preoperative imaging findings, intraoperative assessment, and institutional guidelines designed to minimize bleeding and protect liver function. All patients had normal preoperative liver function, with no evidence of hepatic dysfunction before surgery. The study was approved by the Ethics Committee of the Institution (approval number: 51592021.2.0000.5066). 

### Surgical technique

 All hepatectomies were performed using a standardized approach. In the Pringle group, intermittent hepatic pedicle clamping was performed using a vascular tourniquet. The maneuver consisted of alternating cycles of 15-min maximum ischemia periods followed by 5-min reperfusion intervals. The total Pringle time was calculated as the sum of all ischemia periods, excluding reperfusion intervals. 

### Data collection

 Data were retrieved from electronic medical records and included demographics (age and sex), operative details (surgical time), postoperative outcomes on intensive care unit (ICU) stay, total hospital stay, 30-day mortality, laboratory parameters (bilirubin and prothrombin activity), and long-term outcomes (survival status and survival time in days). Early postoperative liver dysfunction was defined as either bilirubin >2 mg/dL or prothrombin activity <50% within the first seven postoperative days. Follow-up included scheduled clinic visits at 1, 3, 6, and 12 months, then annually, along with laboratory evaluations and imaging studies (computed tomography or magnetic resonance imaging). Survival data were confirmed via active patient follow-up or family contact. 

### Statistical analysis

 All analyses were conducted using Statistical Package for the Social Sciences 20.0 (IBM Corp., Armonk, NY). Continuous data distribution was tested using the Kolmogorov-Smirnov and Shapiro-Wilk tests. Parametric or non-parametric tests were chosen accordingly: Student’s ttest or Mann-Whitney U, respectively. Categorical variables were compared via chi-square or Fisher’s exact test. Overall survival was evaluated through Kaplan-Meier curves and log-rank tests. A Cox proportional hazards model was employed to estimate hazard ratios (HRs) for factors affecting long-term survival. Adjustments were made for potential confounding variables. 

### Bias reduction strategies

 Several strategies were implemented to minimize potential bias. All procedures were performed by a single experienced surgeon to reduce technical variations across cases, while standardized perioperative management protocols were consistently applied. Early liver dysfunction was assessed using predefined criteria (bilirubin >2 mg/dL or prothrombin activity <50% within 7 days) to ensure standardized outcome comparisons. Multivariate analysis adjusted for potential confounding variables, and a specific subgroup analysis of patients with colorectal metastases was conducted to account for pathology-specific variations. Additionally, statistical methods included control for multiple comparisons to minimize type I error in the analysis. 

### Missing data

 All analyses used a complete-case approach once it was confirmed that data were missing at random. Sensitivity analyses did not reveal any significant impact from missing data patterns. 

## RESULTS

Table 1 presents the main demographic and perioperative data. Notably, groups were comparable in age and proportion of major hepatectomies, but Pringle patients showed a higher percentage of males. ICU stay, operative time, and bilirubin levels varied significantly across groups. 

**Table 1:** Baseline characteristics and outcomes of patients undergoing hepatectomy

Characteristic	Pringle (n=96)	Non-Pringle (n=102)	p-value
Age (years)*	59.27±13.16	58.89±12.16	0.834
Male sex, n (%)	53 (55.2)	39 (38.2)	0.017
Major hepatectomy, n (%)	49 (51.0)	44 (43.1)	0.265
Surgical time (min)*	226.87±82.18	184.00±80.90	<0.001
ICU stay (days)*	4.02±2.1	3.11±1.9	0.026
Bilirubin (mg/dL)*	2.18±0.33	3.13±0.39	0.049
Prothrombin activity (%)*	64.4±24.37	64.66±23.35	0.938
30-day mortality, n (%)	11 (11.5)	12 (11.8)	0.514
Overall survival (days)†	1435 (1175–1696)	1026 (790–1262)	0.118

* Values expressed as mean±standard deviation;

† Values expressed as mean (95%CI)

**ICU: intensive care unit**; CI: confidence interval.

### Surgical outcomes

 Patients in the Pringle group experienced a significantly longer operative time (226.87 vs. 184 min, p<0.001) and a slightly extended ICU stay (4.02 vs. 3.11 days, p=0.026). However, total hospital stay did not differ significantly between the two groups (6.83 vs. 7.12 days, p=0.654). 

### Liver function and postoperative course

**Figure 1:**
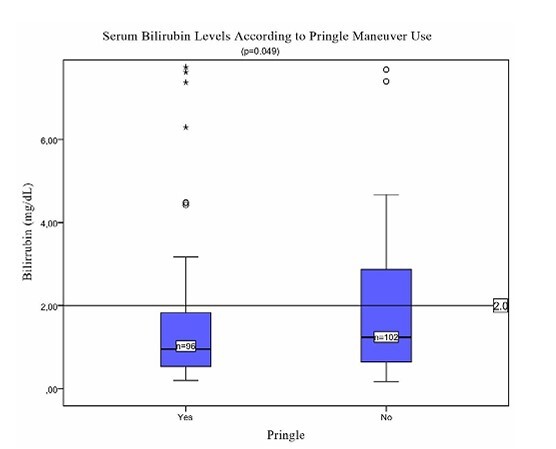
Serum bilirubin levels according to Pringle maneuver use (p=0.049). Boxplot comparing postoperative bilirubin levels between patients who underwent Pringle maneuver (Yes, n=96) versus those who did not (No, n=102). The horizontal line at 2.0 mg/dL represents the threshold for early liver dysfunction.

 Despite normal preoperative liver function in all patients, postoperative bilirubin levels were lower in the Pringle group (2.18±0.33 vs. 3.13±0.39 mg/dL, p=0.049; Figure 1), whereas prothrombin activity was essentially the same (64.4 vs. 64.66%, p=0.938). Thirty-day mortality did not differ between groups (11 vs. 12 patients; 11.5 vs. 11.8%, p=0.514). 

### Survival analysis

**Figure 2:**
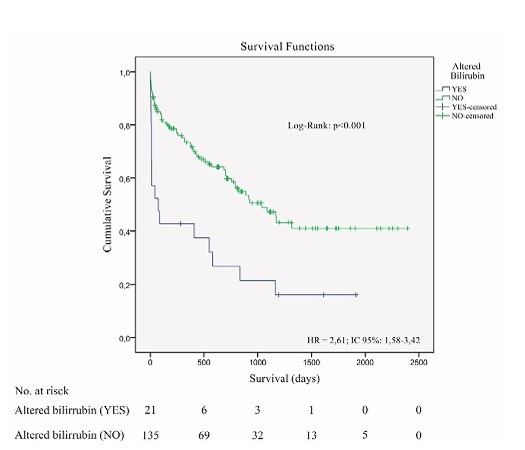
Kaplan-Meier s urvival curves demonstrate that early postoperative liver dysfunction (bilirubin >2 mg/dL within 7 days) is associated with significantly lower survival (p<0.05)

 Although the Pringle group showed a numerically higher average overall survival (1435 vs. 1026 days), the difference was not statistically significant (p=0.118). On the other hand, early postoperative liver dysfunction — defined by bilirubin >2 mg/dL within 7 days — was an intense predictor of worse survival (510 vs. 1270 days, HR 2.61, 95% confidence interval [95%CI] 1.58–3.42, p<0.001, Figure 2). Kaplan-Meier curves demonstrated early and persistent separation, underscoring the prognostic weight of early bilirubin elevation. 

### Duration of pringle maneuver

 When analyzing Pringle times, four duration brackets emerged (n=96): 15 min: 30 (31.6%), 16–30 min: 34 (35.8%), 31–45 min: 22 (23.2%), and >45 min: 9 (9.5%). Survival analysis by these time brackets indicated marginal statistical significance (log rank p=0.054, Breslow p=0.018, Tarone-Ware p=0.026), with the 16–30-min group presenting the most favorable mean survival (1580 days, 95%CI 1179–1981). 

### Colorectal metastases subgroup

 Among patients with colorectal liver metastases (n=74), no notable differences emerged regarding overall survival (1181 vs. 811 days, p=0.384), ICU stay (3.34 vs. 2.83 days, p=0.147), or hospital stay (6.5 vs. 6.43 days, p=0.532). These findings hint that the maneuver’s influence may be nuanced by pathology-specific factors. 

### Multivariate analysis

**Figure 3:**
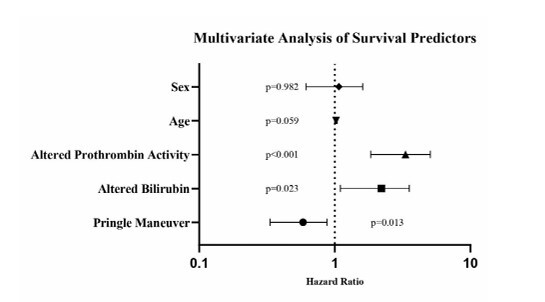
Multivariate analysis of survival predictors. Forest plot showing hazard ratios and 95% confidence intervals for factors affecting long-term survival. The vertical dotted line represents HR 1.0. Values to the right indicate increased risk, while values to the left indicate protective effects. p-values are shown for each variable.

 Multivariate Cox regression analysis identified three key independent survival predictors (Table 2, Figure 3). The Pringle maneuver demonstrated a protective effect with reduction in mortality risk (HR 0.540, 95%CI 0.333–0.876, p=0.013). Early postoperative liver dysfunction markers strongly predicted worse outcomes: elevated bilirubin (>2 mg/dL within 7 days) was associated with nearly doubled mortality risk (HR 1.975, 95%CI 1.100–3.545, p=0.023), while decreased prothrombin activity showed a threefold increase in mortality risk (HR 3.055, 95%CI 1.839–5.075, p<0.001). Age showed a trend toward significance (HR 1.021, 95%CI 0.999–1.043, p=0.059), while sex did not significantly affect outcomes (HR 0.994, p=0.982). Analysis of different Pringle duration groups revealed significant variations in surgical time (p=0.002), with the 16–30 min group showing the shortest surgical time (200.97 min) compared to >45 min (309.17 min). While prothrombin activity showed differences among these groups (p=0.043), bilirubin levels were not significantly different (p=0.105). 

**Table 2:** Multivariate Cox regression analysis of factors affecting long-term survival after hepatectomy.

Variable	Hazard ratio	95%CI	p-value
Pringle Maneuver	0.540	0.333–0.876	0.013
Altered bilirubin	1.975	1.100–3.545	0.023
Altered prothrombin activity	3.055	1.839–5.075	<0.001
Age	1.021	0.999–1.043	0.059
Sex	0.994	0.615–1.608	0.982

CI: confidence interval.

## DISCUSSION

 This comprehensive analysis demonstrates the protective role of the intermittent Pringle maneuver in modern hepatic surgery^7^ . Using cycles of 15-min maximum ischemia followed by 5-min reperfusion periods, our multivariate analysis revealed that the maneuver strongly reduces mortality risk (HR 0.540, p=0.013). This finding aligns with the lower immediate bilirubin levels observed in the Pringle group, suggesting that controlled ischemia-reperfusion periods allow for more precise parenchymal transection while minimizing cumulative blood loss. 

 Crucially, our results reinforce that early postoperative liver dysfunction stands out as a powerful prognostic factor. Patients who developed bilirubin >2 mg/dL or prothrombin activity <50% within the first postoperative week exhibited significantly increased mortality risks (HR 1.975, p=0.023 and HR 3.055, p<0.001, respectively). These findings emphasize the clinical importance of close surveillance of liver function markers, particularly coagulation parameters, in the early postoperative window. 

 Interestingly, when we explored the total duration of intermittent clamping, an interval of 16–30 min appeared most favorable, suggesting that this balanced approach to vascular control may optimize bleeding control without excessive cumulative ischemia-reperfusion damage. Combined with our standardized protocol of 15-min maximum ischemia cycles, these findings warrant a more personalized strategy for vascular control based on individual patient factors. 

 Limitations of this study include its retrospective nature and the single-surgeon context, which may restrict generalizability. Although all patients had preserved preoperative liver function, the heterogeneous nature of surgical indications, with colorectal metastases predominating, may have influenced outcomes differently across subgroups. Additionally, the study’s single-center design could have introduced selection bias. However, the standardized approach across all hepatectomies and consistent perioperative management likely minimized confounding from technique variability. 

 Concluding, the intermittent Pringle maneuver demonstrated a significant protective effect on survival (HR 0.540, 95%CI 0.333–0.876, p=0.013). A total clamping duration of 16–30 min appeared most advantageous. Early postoperative liver dysfunction emerged as a strong predictor of poor outcomes, with altered bilirubin (>2 mg/dL) nearly doubling mortality risk (HR 1.975, 95%CI 1.100–3.545, p=0.023) and decreased prothrombin activity (<50%) tripling it (HR 3.055, 95%CI 1.839–5.075, p<0.001) within the first postoperative week. These findings emphasize that while the controlled intermittent Pringle maneuver offers survival benefits, careful monitoring of postoperative liver function remains crucial for long-term outcomes. 

## CONCLUSIONS

 The intermittent Pringle maneuver, in this retrospective cohort of 198 hepatectomies, using 15-min ischemia/5-min reperfusion cycles, was associated with longer operative times and increased ICU stays, while early postoperative liver dysfunction demonstrated a strong predictor of poor outcomes. Future prospective trials and larger multi-institutional studies would be beneficial in confirming our observations, refining optimal Pringle times for different clinical scenarios, and establishing clearer guidelines for the maneuver’s application in specific scenarios. 
